# External Validation of the SERC Trial Population: Comparison with the Multicenter French Cohort, the Swedish and SENOMIC Trial Populations for Breast Cancer Patients with Sentinel Node Micro-Metastasis

**DOI:** 10.3390/cancers12102924

**Published:** 2020-10-11

**Authors:** Gilles Houvenaeghel, Houssein El Hajj, Julien Barrou, Monique Cohen, Pédro Raro, Jérémy De Troyer, Pierre Gimbergues, Christine Tunon de Lara, Vivien Ceccato, Véronique Vaini-Cowen, Christelle Faure-Virelizier, Frédéric Marchal, Tristan Gauthier, Eva Jouve, Pierrick Theret, Claudia Regis, François Desmons, Agnès Tallet, Jean-Marie Boher, the SERC Trial Group

**Affiliations:** 1 Department of Surgical Oncology, Institut Paoli Calmettes & CRCM & Aix Marseille Univ, 232 Bd de Sainte Marguerite, 13009 Marseille, France; houvenaeghelg@ipc.unicancer.fr (G.H.); barrouj@ipc.unicancer.fr (J.B.); cohenm@ipc.unicancer.fr (M.C.); talleta@ipc.unicancer.fr (A.T.); boherjm@ipc.unicancer.fr (J.-M.B.); 2 Institut de Cancérologie de l’Ouest—Site Paul Papin, 15 rue André Boquel, 10059 49055 Angers CEDEX 02, France; Pedro.raro@ico.unicancer.fr; 3 Polyclinique Urbain V, Chemin du Pont des Deux Eaux, 84000 Avignon, France; detroyerjeremie@gmail.com; 4 Department of Surgical Oncology, Centre Jean Perrin, 58 rue Montalembert BP 392, 63011 Clermont Ferrand CEDEX, France; Pierre.GIMBERGUES@clermont.unicancer.fr; 5 Department of Surgical Oncology, Institut Bergonie, 229 cours de l’Argonne, 33076 Bordeaux CEDEX, France; C.TunondeLara@bordeaux.unicancer.fr; 6 Department of Surgical Oncology, Institut Jean Godinot, 1 rue du Général Koenig, 51056 Reims, France; vivien.ceccato@reims.unicancer.fr; 7 Department of Gynecology and Obstetrics, Clinique du Parc Rambot 2, Avenue du Dr Aurientis, 13100 Aix en Provence, France; vainicowen@orange.fr; 8 Department of Surgical Oncology, Centre Léon Bérard, 28 rue Laennec, 69373 Lyon CEDEX 8, France; christelle.faure@lyon.unicancer.fr; 9 Department of Surgical Oncology, Centre Alexis Vautrin, 6 Avenue de Bourgogne, 54519 Vandœuvre-lès-Nancy, France; f.marchal@nancy.unicancer.fr; 10 Department of Gynecology and Obstetrics, Hopital de la mère et de l’enfant, 8 Avenue Dominique Larrey, 87000 Limoges, France; Tristan.Gauthier@chu-limoges.fr; 11 Department of Surgical Oncology, Institut Universitaire du Cancer Toulouse, Oncopole, 1 Avenue Irène Joliot-Curie, 31059 Toulouse, France; jouve.eva@iuct-oncopole.fr; 12 Department of Gynecology and Obstetrics, CH Saint Quentin, 1 avenue Michel de l’Hospital, B.P. 608, 02321 Saint Quentin CEDEX, France; theret.pierrick@chu-amiens.fr; 13 Department of Senology, Centre Oscar Lambret, 3 rue F. Combemal, 59000 Lille, France; c-regis@o-lambret.fr; 14 Department of Gynecology and Obstetrics, GHM de Grenoble, La Clinique des Eaux Claires, 8 rue du Dr Calmette, 38028 Grenoble CEDEX 1, France; francoisdesmons@hotmail.com; 15 Department of Radiotherapy, Institut Paoli Calmettes & CRCM & Aix Marseille Univ, 232 Bd Ste Marguerite, 13009 Marseille, France; 16 Department of Biostatistics, Institut Paoli Calmettes & CRCM & Aix Marseille Univ, 232 Bd Ste Marguerite, 13009 Marseille, France

**Keywords:** breast cancer, sentinel node, micro-metastases, trial

## Abstract

**Simple Summary:**

After the results of many trials, it is now accepted to omit axillary dissection in selected patients with limited axillary involvement. However, the external validity of these trials is questionable. Our study aimed to evaluate the accuracy of the real French population representativity in the SERC (Sentinelle Envahi et Randomisation du Curage) trial population for patients with breast cancer (BC) associated with sentinel node (SN) micro-metastasis and the differences between the studied population and the real French population. The secondary aim was to compare the French and the Swedish populations of patients with SN micro-metastasis. The findings of our study in addition to the previously demonstrated concordance between the SENOMIC (Sentinelle node Micrometastasis) trial and the Swedish National Breast Cancer Registry (NKBC) populations implied that the results of both the SERC and the SENOMIC trials can be applied to both the French and Swedish real populations.

**Abstract:**

Many trials confirmed the safety of omitting axillary dissection in the selected patients treated for early breast cancer. The external validity of these trials is questionable. Our study aimed to evaluate the accuracy of the French population representativity in the SERC trial and the differences between these two populations as well as comparing the French and the Swedish populations (the SENOMIC trial population and the Swedish National Breast Cancer Registry (NKBC) cohort) of patients with sentinel node (SN) micro-metastasis. A higher rate of smaller tumors and grade 1 tumors was observed in the French cohort when compared to the SERC population. Our findings conclude that both French populations show similar characteristics. Positive non-sentinel node (NSN) rates at completion axillary lymph node dissection (ALND) were 10.28 % and 11.3 % in the SERC trial and French cohort, respectively (*p* = 0.5). The rate of grade 1 tumors was lower in the SENOMIC trial (16.2%) and in the NKBC cohort (17.4%) compared to the SERC trial population (27.3%) and the French cohort (34.4%). Our findings in addition to the previously demonstrated concordance between the SENOMIC trial and the NKBC populations imply that the results of both the SERC and the SENOMIC trials can be applied to both French and Swedish real populations.

## 1. Introduction

Modern medicine relies mainly on randomized controlled trials (RCTs) and systematic reviews to guide the treatment plan. All studies and trials ensure that internal validity is respected by eliminating biasing factors [1]. In order to apply the results of clinical trials to the general population, external validity should be ensured. In 2016, Ford and Norrie discussed the tendency to select the healthiest patients to include in the trials [2].

In breast cancer management, lymph node (LN) involvement is considered as one of the most important prognostic factors [3]. Axillary treatment in breast cancer (BC) patients includes axillary lymph node dissection (ALND) and axillary radiation therapy (ART). The significant short-term and long-term morbidity associated with the axillary treatment led to many trials evaluating its necessity in BC management [4,5]. During the last three decades, and following many prospective randomized controlled trials, axillary treatment witnessed important milestones and especially a trend towards therapeutic de-escalation. The results of the NSABP-B-32 trial showed that in patients with early BC and negative sentinel nodes, ALND can be omitted and SLNB (sentinel lymph node biopsy) can be safely performed with no impact on the overall and disease-free survival or regional control [6]. Following this trial, the ACOSOG-Z011 and the IBCSG-23-01 trials with their corresponding 10 year follow-up and 5 year follow-up, respectively, showed that it was safe to omit ALND in patients presenting early BC with one or two positive SNs without extra capsular extension treated conservatively. The ACOSOG-Z011 criteria to omit ALND include patients having fewer than two macro-metastases (>2 mm) while the IBCSG-23-01 trial included patients with only micro-metastases (≤2 mm) or isolated tumor cells (ITC: (≤0.2 mm)) [7,8,9,10]. Both of the above cited trials induced important changes in clinical practice by eliminating the indications of ALND in patients presenting micro-metastases and patients presenting less than two SLN macro-metastases; however, the extrapolation of these results to the general population has been questioned. Both trials closed prematurely [11], did not achieve full patient accrual, presented lower numbers of events compared to what was expected, and the majority of patients included had favorable tumor prognostic features [4,12,13,14,15,16].

The above cited trials were not designed to specifically evaluate patients presenting SN micro-metastasis. The ACOSOG-Z011 trial did not differentiate patients with micro-metastases from patients with macro-metastases. The IBCSG-23-01 trial did not differentiate between ITC and micro-metastasis. However, the AATRM 048/13/2000 trial included 233 patients and was designed to only evaluate patients with SN micro-metastases [17]. Both the IBCSG-23-01 and the AATRM 048/13/2000 trials included low numbers of patients who underwent mastectomy: 86 (9.2%) patients and 18 patients, respectively [10,17].

The SERC and the SENOMIC trials were designed with the intention of confirming the safety of the cALND omission in the populations of patients under represented in previously published trials. These populations include patients having mastectomy or patients presenting less favorable prognostic characteristics [12,18,19].

The SERC trial is a French multicentric prospective randomized non-inferiority phase 3 trial aiming to compare complementary axillary lymph node dissection (cALND) versus no cALND in patients with BC and SN metastases. Patients are stratified according to their axillary involvement (ITC, micro-metastasis and macro-metastasis) [19].

The SENOMIC trial is a prospective trial aiming to confirm the safety of ALND omission in the case of SLN micro-metastases in patients undergoing conservative treatment or mastectomy and in patients with less favorable prognostic characteristics. Andersson et al. compared the cohort of this study to the cohort extracted from the Swedish National Breast Cancer Registry (NKBC) during the same period of time [12].

Since the most frequent criticism of all trials is the lack of external validity, clinical trials should be designed in a way to allow clinicians to apply the results to the general population. Therefore, the primary aim of our study was to evaluate the external validity of the SERC trial by evaluating the accuracy of the real French population representatively in the SERC trial population for patients with BC associated with SN micro-metastasis and the differences between the studied population and the real French population. The secondary aim was to compare the French and the Swedish populations of patients with BC with SN micro-metastasis.

## 2. Results

### 2.1. Comparisons between SERC Trial and French Cohort Populations

Table 1 shows the characteristics of patients in both the SERC and the French cohort. Age distribution was significantly different between the two cohorts (*p* < 0.05). More patients with pT1 stages and more patients with grade 1 tumors were found in the French cohort (*p* = 0.001) and (*p* < 0.01), respectively. The number of patients who had two or less SLNBs was higher in the SERC trial (*p* < 0.001). More patients underwent internal mammary chain radiation (IMR) in the French cohort (*p* < 0.001). Trastuzumab treatment was more prevalent in the SERC population (11.55%) compared to (6.05%) the French cohort (*p* < 0.01). Patients eligible to the criteria of the ACOSOG-Z011 trial were more prevalent in the French cohort (*p* < 0.001) and this is in accordance with the main objective of the trial aiming to evaluate the safety of omitting cALND in patients undergoing mastectomies and extra-capsular extension.

No significant difference was found between the two groups concerning the tumor histology, lympho-vascular invasion (LVI), endocrine receptors, Her2 status, tumor phenotypes, number of positive SNs, chemotherapy, surgery, endocrine therapy, radiotherapy (breast or post-mastectomy radiation therapy (PMRT)), clavicular radiation and non-sentinel node (NSN) status at cALND. Positive NSN rates at cALND were 10.28% and 11.3% in the SERC trial and French cohort, respectively (*p* = 0.5). The SERC trial recruited patients between 2012 and 2018 while the French cohort population included patients between 2009 and 2012.

### 2.2. Comparisons between French and Swedish Populations

As shown in Table 2, when comparing the SERC trial population, the SENOMIC trial population and the NKBC population, significant differences were noted: the SERC trial population presented the lowest rates of grade 2 and grade 3 tumors (*p* < 0.001) and the lowest rate of mastectomies (*p* < 0.0001). The SERC trial population had the highest rate of adjuvant radiation therapy (*p* < 0.0001). There was no significant difference in the rate of chemotherapy administration between the SENOMIC and the NKBC on one side, neither between the SERC and the SENOMIC on the other side, but when comparing the patients from the SERC trial and the NKBC trial, we found a significant difference with a higher rate of chemotherapy administration in the SERC trial (*p* < 0.05).

All patients included in the SENOMIC trial underwent SLNB alone compared to 62.4% in the NKBC cohort and 54.04% in the SERC trial population ((*p* < 0.01) when comparing the NKBC and the SERC populations).

No significant difference was found for the other analyzed factors: tumor histology, estrogen and progesterone receptors, Her2 status, endocrine therapy and Trastuzumab. Regional nodal irradiation (RNI) was not performed in the SENOMIC trial in comparison with IMR and clavicular radiotherapy in 35.65% and 56.43%, respectively, in SERC trial.

Median ages were 58.0, 56.0, 62.0 and 63.0 years old for SERC trial, the French cohort, SENOMIC trial and NKBC, respectively, and the mean tumor sizes were 18.73 mm, 18.1, 18 and 19 mm, respectively.

The periods of the SERC trial and Swedish studies (SENOMIC trial and NKBC cohort) were 2012–2018 and 2014–2017, respectively.

## 3. Discussion

After the results of both the ACOSOG Z0011 and the IBCG 23-01 trials, it is considered safe to omit ALND in SLN positive patients. This had a major impact on clinical practice in breast cancer management and ALND omission was generalized even to the populations of patients under represented in these trials [8,9,13]. The use of restrictive inclusion/exclusion criteria in RCTs was identified as being one of the key factors limiting the external validity of trial findings [20]. Kennedy-Martin et al. concluded in their literature review on external validity that patients with poor disease prognosis in oncology studies were usually excluded. This was applicable to the previously cited trials since patients with less favorable prognostic characteristics, or those undergoing mastectomy, were under represented [20]. In order not to risk the under-treatment of under represented subgroups, it is important to ensure external validity and that trial outcomes are generalizable to patients that are treated in the general clinical setting. RCT trial populations are often highly selected and represent a population at much lower risk of negative events and complications. The strive to ensure the homogeneity of the study population enhances internal validity by removing the confounding factors and reducing differences between the patients. Reducing bias and confounding factors help make an association between the treatment and the outcome but has a negative effect on external validity [20].

In the ACOSOG Z011, inequalities between patients were in the favor of the SLN group with a higher rate of micro-metastases in the SLN group. The tumor burden in the study was lower than anticipated and the patients in real life who meet the eligibility criteria have higher chances of having additional positive nodes [15]. Axillary recurrences were not an endpoint and a significant amount had unknown nodal status [4].

The characteristics in the French cohort and the SERC trial were compared for the patients presenting breast cancer with sentinel lymph node biopsy and a confirmed SN micro-metastasis. Only a few significant differences were observed when comparing the SERC and the French cohorts. A minor difference between the two groups was the presence of a higher rate of smaller (pT1) and grade 1 tumors in the French cohort compared to the SERC cohort (*p* < 0.01). Another minor difference was noted concerning the Her2 status determination and Trastuzumab treatment that was more prevalent in the SERC trial. This reflects the endorsement of Trastuzumab treatment for Her2 positive patients in clinical guidelines after 2005. The higher rate of “less than two SLN” in the SERC trial cohort was considered a major difference reflecting the improvement in SLN detection techniques and the increased confidence of the surgeons in this technique after it proved its efficiency. These findings show that both these populations have similar characteristics.

Out of the three trials evaluating the role of axillary treatment in the case of axillary involvement, the ACOSOG-Z0011 trial included 35.1% patients with micro-metastases but in the analysis no differentiation was made between the patients with micro-metastases and patients with macro-metastases [13]. This issue was addressed in the IBCSG-23-01, where the recruited patients had only micro-metastases. However, no differentiation was made between the micro-metastases and ITC [8]. The AATRM 048/13/2000 trial included only patients with micro-metastases but the cohort included a small number of patients (*n* = 233) [17].

When comparing the tumor sizes in the different trials, we find that the average tumor size is relatively similar between the different cohorts. The rate of T1 tumors was the lowest in the SENOMIC trial cohort (63%) compared to 63.46% in the SERC trial cohort, 67.9% in the French cohort, 69% in the ACOSOG Z0011 trial cohort and the IBCSG 23-01 trial cohort and 72.6% in the AATRM 048/13/2000 trial cohort [17].

The rate of grade 1 tumors was also lower in the SENOMIC trial (16.2%) and in the NKBC cohort (17.4%) compared to previous trials (24% in ACOSOG Z0011 and 22% in IBCSG 23-01, 74.07% in AATRM trial) and compared to the SERC (27.3%) and French cohort (34.4%) trial.

A lower rate of mastectomies and a lower rate of grade 2–3 tumors were observed in the SERC trial compared to the NKBC cohort and the SENOMIC trial cohort, even though they were performed in the same period of time. The mastectomy rate in the IBCSG-23-01 trial [8] was 9.2% (86 patients), which included patients with isolated tumor cells (ITC) and micro-metastases without a distinct analysis between these two types of SN involvement. In the AATRM trial [17] dedicated to SN micro-metastases, the mastectomy rate was 7.7% (18/233). The mastectomy rates were higher in the SERC and SENOMIC trials as well as in the French and Swedish cohorts, ranging from 14.7% in the French cohort to 43.6% in the NKBC cohort and an overall average of 23.76%. The higher rate of mastectomies in the SENOMIC and NKBC populations was considered a minor difference and is probably due to the wider application of conservative and oncoplastic surgery in France. This might also explain the higher rate of adjuvant radiation therapy in the SERC trial, another minor difference.

Positive NSN rates after cALND were comparable between the different trials with a rate of 13% (15/112) in the AATRM-trial, 11.3% in the French cohort and 10.28% in the SERC trial.

In a previous publication, we reported results on paired numbers of patients with SN micro-metastases from the French cohort. The results were in favor of cALND compared to no cALND in patients undergoing mastectomy for BC with SN micro-metastases [21]. The results of the ongoing SERC and SENOMIC trials were therefore awaited in order to provide sufficient evidence to suggest the omission of cALND.

The other ongoing trials, POSNOC, INSEMA, SENOMAC and SINODAR trials [3,4,22,23] include breast cancer patients presenting macro-metastases only and undergoing conservative treatment or mastectomy. These trials randomize patients for the performance or omission of axillary dissection.

To our knowledge, there is only one study that analyzed the representativity of the real population in the cohorts of the trials evaluating the safety of de-escalating axillary treatment. Andersson et al. found an acceptable concordance between the SENOMIC trial cohort and the NKBC population [12]. These findings and the findings of our study mean that the results of the SENOMIC trial and the results of the SERC trial can be applied to real population patients. In clinical practice, it is important to consider the differences between the different cohorts of patients.

## 4. Materials and Methods

### 4.1. Populations

The SERC population patients were recruited from 53 institutions over an accrual period of 73 months (from July 2012 to July 2018). An average of 309 patients were recruited every year. A total number of 1855 patients were included in the trial. This trial was registered under the number NCT01717131 in the ClinicalTrials.gov. The protocol was approved by the institutional review boards of all participating centers (CPP approval reference: 1226, approved on the 09 May 2012) and all participants provided written informed consent. Eligibility criteria included tumors ≤cT2N0, multi-centric tumors, one or more positive SNs, ITC, micro-metastases, macro-metastases, with or without capsular effraction, breast conserving surgery (BCS), mastectomy, neo-adjuvant chemotherapy (NAC) with SLNB performed prior to the NAC. Exclusion criteria included previous or concomitant malignancy, pure ductal carcinoma in situ, previous systemic therapy before SLNB, distant metastases and clinically suspicious axillary nodes [19].

Whole breast irradiation (WBI) associated with a boost on the tumor basin was recommended for all patients undergoing CBS. Post-mastectomy radiation therapy (PMRT) was proposed as indicated by the (regional) guidelines and was either started 4 to 8 weeks after surgery or after the completion of adjuvant chemotherapy (AC). ART was not performed; instead, two tangential fields were recommended in the case of chest irradiation or WBI with a total dose of 50 grays delivered in 25 fractions of 2 grays over a period of 5 weeks. AC and endocrine therapy (ET) were proposed according to the (regional) guidelines followed by each center.

SN detection was either performed using the radioisotope method alone or in combination with the blue dye colorimetric method. Injections of the blue dye or radioisotope were performed either in the peri-tumoral region or in the retro-areolar region.

Pathologic examination of the SN included sections of the entire SN at 50–200 μm intervals and all sections were examined with hematoxylin and eosin staining (HES). Other methods included cytokeratin immunostaining that was performed when HES staining was negative, one step nucleic acid amplification. A CK19 mRNA copy number between 250 and 5000 copies/μL was considered as micro-metastasis, and a copy number greater than 5000 copies/μL was considered as macro-metastases. Out of this population, 471 patients with SN micro-metastases were extracted and included in this study.

The French population cohort included all patients treated for early BC with cN0 and SLNB in any of the 15 cancer centers in France between March 1999 and March 2012 [24]. Out of the 23,000 patients treated for breast cancer included in the French cohort, we included all 1863 patients who had SN micro-metastases in this study (an average of 143 patients per year). In all centers, the SN detection techniques as well as the SN pathologic analysis techniques were identical to the ones performed in the SERC trial. Only patients who underwent neo-adjuvant treatment were excluded from the study.

Most of the centers that included patients in the French cohort between March 1999 and March 2012, participated in the patient’s accrual for the SERC trial after 2012.

The SENOMIC trial population included 548 patients treated for primary BC with cN0 and SN micro-metastases between January 2014 and March 2017 (an average of 169 patients per year), all of which were treated in any of the 23 included Swedish centers. Patients underwent either conservative surgery or mastectomy but none of them underwent cALND or regional nodal irradiation (RNI). This trial did not include patients who had T3 tumors or patients who underwent NAC [12].

The NKBC cohort includes data on 585 breast cancer patients with SN micro-metastases who were diagnosed between January 2014 and March 2017. These data were extracted from the Swedish National Breast Cancer Registry (NKBS) [12]. Patients with more than two tumors and patients receiving NAC were excluded from this cohort. In this cohort, it was not specified whether RNI was included in the radiation field or not.

### 4.2. Statistical Analysis

Patient and tumor characteristics are presented as distributions with their percentages (categorical data) or median values within their range (continuous data). The distribution of categorical data was tested by the chi-square test and that of continuous data by the Student’s t test.

The statistical analysis was performed using SPSS software (IBM Corp., Armonk, N.Y., USA) version 24, and statistical significance was set at *p* < 0.05 for all tests.

We first compared the SERC trial population to the French cohort population. Then, we compared the French and Swedish populations.

## 5. Conclusions

The findings of our study in addition to the previously demonstrated concordance between the SENOMIC trial and the NKBC populations imply that the results of both the SERC and the SENOMIC trials can be applied to both French and Swedish real populations.

## Figures and Tables

**Table 1:** Comparison of the SERC trial and the French cohort (G3S) populations.

Sentinel Node Micro-Metastases	Characteristics	SERC		G3S		Chi 2
		***n***	**%**	***n***	**%**	***p*-value**
All patients		471		1863		
Age	≥40	24	5.10	135	7.30	<0.05
	41–75	432	91.72	1632	87.60	
	>75	15	3.18	95	5.10	
	Missing data	0		1		
	Median (range)	58 (26–83)		56 (25–94)		
pT stage	pT1	297	63.46	1266	67.90	0.001
	pT2	155	33.12	522	28.00	
	pT3	6	1.28	55	3.00	
	pT4	2	0.43			
	Missing data	11		20	1.10	
pT size	≤10	87	18.59	453	24.60	<0.01
	10 to 30	334	71.37	1256	68.30	
	>30	47	10.04	130	7.10	
	Missing data	3		24		
Tumor size	Mean	18.73		18.1		
T histology	Ductal	373	79.36	1515	81.30	0.40
	Lobular	47	10.00	173	9.30	
	Mixed	15	3.19	39	2.10	
	Others	35	7.45	119	6.40	
	Missing data	1		17	0.90	
Grade	1	125	27.29	641	34.40	<0.01
	2	234	51.09	872	46.80	
	3	99	21.62	308	16.50	
	Missing data	13		42	2.30	
LVI	No	334	74.22	1192	64.00	>0.05
	Yes	116	25.78	516	27.70	
	Missing data	21		155	8.30	
ER	Negative	44	9.50	148	8.20	0.40
	Positive	419	90.50	1661	91.80	
	Missing data	8		54		
PR	Negative	89	19.18	388	22.70	0.15
	Positive	375	80.82	1321	77.30	
	Missing data	7		154		
Endocrine Receptors	Negative	44	9.48	129	6.90	>0.05
	Positive	420	90.52	1707	91.60	
	Missing data	7		27	1.40	
Her2	Negative	403	89.56	1100	95.60	0.15
	Positive	47	10.44	95	4.40	
	Missing data	21		668		
T phenotype	ER+ Her2+	37	8.24	67	5.60	0.20
	ER+ Her2-	373	83.07	1034	86.70	
	ER- Her2+	10	2.23	28	2.30	
	ER- Her2-	29	6.46	64	5.40	
	Missing data	22		670		
SN number	≤2	344	73.04	940	63.00	<0.001
	>2	127	26.96	551	37.00	
	Missing data	0		372		
Positive SN number	≤2	465	99.15	1481	99.30	0.60
	>2	4	0.85	10	0.70	
	Missing data	2		372		
pN status final	pN1mi	198	95.19	1710	91.80	0.09
	pN1macro	10	4.81	153	8.20	
	Missing data					
Chemotherapy	No	202	43.53	761	40.80	0.80
	NAC (Neoadjuvant chemotherapy)	11	2.37	37	2.00	
	AC (Adjuvant chemotherapy)	251	54.09	992	53.20	
	Missing data	7		73	3.90	
Surgery	Conservative	388	82.55	1554	83.40	0.18
	Mastectomy	82	17.45	274	14.70	
	Missing data	1		35	1.90	
Endocrine therapy	No	40	10.13	199	11.10	0.50
	Yes	355	89.87	1594	88.90	
	Missing data	76		70		
Trastuzumab	No	291	88.45	1118	93.50	<0.01
	Yes	38	11.55	77	6.05	
	Missing data	142			668	
Radiotherapy	No	27	5.87	113	6.50	0.50
	Yes	433	94.13	1636	93.50	
	Missing data	11		114		
Internal Mammary Chain radiotherapy	No	269	64.35	805	51.40	<0.001
	Yes	149	35.65	761	48.60	
	Missing data	53		297		
Clavicular radiotherapy	No	183	43.57	751	48.00	0.10
	Yes	237	56.43	814	52.00	
	Missing data	51		298		
Treatment realized	ALND	216	45.96	1588	85.20	<0.0001
	SLNB	254	54.04	275	14.80	
Eligibility Z0011	No	119	27.67	126	9.30	<0.0001
	Yes	311	72.33	1226	90.70	
	Missing data	41				
NSN status at cALND	pN0	192	89.72	1188	88.70	0.50
	pN1macro	22	10.28	152	11.30	

**Table 2:** Comparison of the French and the Swedish populations.

SN Micro-Metastases	Characteristics	SERC		French		Chi 2	SENOMIC		NKBC		SENOMIC	SERC Versus	SERC
				**Cohort**							**Versus NKBC**	**SENOMIC**	**Versus NKBC**
		***n***	**%**	***n***	**%**	***p*-Value**	***n***	**%**	***n***	**%**	***p*-Value**	***p*-Value**	***p*-Value**
All patients		471		1863			548		585				
	Median (range)	58 (26–83)		56 (25–94)			62 (28–90)		63 (28–95)				
Tumor size	Mean	18.73		18.1			18		19		0.026		
T histology	Ductal	373	79.36	1515	81.30	0.40	448	81.70	482	82.40	0.921	0.15	0.25
	Lobular	47	10.00	173	9.30		63	11.50	60	10.30			
	Mixed	15	3.19	39	2.10		12	2.20	16	2.70			
	Others	35	7.45	119	6.40		25	4.60	27	4.60			
	Missing data	1		17	0.90								
Grade	1	125	27.29	641	34.40	><0.01	89	16.20	101	17.40	0.655	<0.001	<0.001
	2	234	51.09	872	46.80		311	56.80	310	53.40			
	3	99	21.62	308	16.50		144	26.30	158	27.20			
	Missing data	13		42	2.30		4	0.70	12	2.00			
ER	Negative	44	9.50	148	8.20	0.40	49	8.90	54	9.20	0.844	0.90	0.90
	Positive	419	90.50	1661	91.80		499	91.10	528	90.30			
	Missing data	8		54			0		3	0.50			
PR	Negative	89	19.18	388	22.70	0.15	113	20.60	117	20.00	0.828	0.50	0.60
	Positive	375	80.82	1321	77.30		434	79.20	464	79.30			
	Missing data	7		154			1	0.20	4	0.70			
Her2	Negative	403	89.56	1100	95.60	0.15	479	87.40	496	84.80	0.350	0.60	0.20
	Positive	47	10.44	95	4.40		61	11.10	75	12.80			
	Missing data	21		668			8	1.50	14	2.40			
Chemotherapy	No	202	43.53	761	40.80	0.80	258		294	50.30	0.062	0.25	<0.05
	NAC	11	2.37	37	2.00								
	AC	251	54.09	992	53.20		290	52.90	291	49.70			
	Missing data	7		73	3.90								
Surgery	Conservative	388	82.55	1554	83.40	0.18	344	62.80	329	56.20	0.055	<0.0001	<0.0001
	Mastectomy	82	17.45	274	14.70		204	37.20	255	43.60			
	Missing data	1		35	1.90		0		1	0.20			
Endocrine therapy	No	40	10.13	199	11.10	0.50	51	9.50	70		0.245	0.80	0.40
	Yes	355	89.87	1594	88.90		497	90.50	515	88.00			
	Missing data	76		70									
Trastuzumab	No	291	88.45	1118	93.50	<0.01	492	89.80	520	88.90	0.627	0.50	0.80
	Yes	38	11.55	77	6.05		56	10.20	65	11.10			
	Missing data	142			668		0		0				
Radiotherapy	No	27	5.87	113	6.50	0.50	152	27.90	185	31.60	0.239	<0.0001	<0.0001
	Yes	433	94.13	1636	93.50		396	72.10	400	68.40			
	Missing data	11		114			0		0				
Treatment realized	ALND	216	45.96	1588	85.20	<0.0001	0	0	220	37.60	<0.001		<0.01
	SLNB	254	54.04	275	14.80		548	100	365	62.40			
